# Major and minor criteria for gastric dystemperaments in Persian Medicine: Sari gastric dystemperament criteria-I (SGDC-I)

**DOI:** 10.22088/cjim.13.4.681

**Published:** 2022

**Authors:** Mahshid Chaichi-Raghimi, Reza Ilkhani, Elham Parsa, Mahmood Khodadoost, Rasool Choopani, Roshanak Mokaberinejad, Mojgan Tansaz, Mina Movahhed, Hasan Namdar, Elham Emaratkar, Mahdi Alizadeh Vaghasloo, Mamak Hashemi, Malihe Tabarrai, Reihaneh Moeini, Narjes Gorji, Abbas Alipour, Parisa Jafari, Fatemeh Hakimi, Farideh Yaghmaei, Armin Zareiyan, Ali Montazeri, Morteza Mojahedi

**Affiliations:** 1 Department of Traditional Medicine, School of Traditional Medicine, Shahid Beheshti University of Medical Sciences, Tehran, Iran; 2 Student Research Committee, School of Traditional Medicine, Shahid Beheshti University of Medical Sciences, Tehran, Iran; 3 Department of Iranian Traditional Medicine (Persian Medicine), Tehran Medical Sciences, Islamic Azad University, Tehran, Iran; 4 Traditional Medicine and Materia Medica Research Center, Shahid Beheshti University of Medical Sciences, Tehran, Iran; 5 Traditional Medicine Clinical Trial Research Center, Shahed University, Tehran, Iran; 6 Department of Traditional Medicine, School of Medicine, Shahed University, Tehran, IR Iran; 7 Department of Traditional Medicine, School of Persian Medicine, Tehran University of Medical Sciences, Tehran, Iran; 8 Persian Medicine Network (PMN), Universal Scientific Education and Research Network (USERN), Tehran, Iran; 9 Department of Persian Medicine, School of Medicine, Hamadan University of Medical Sciences, Hamadan, Iran; 10 Department of History of Medical Sciences, School of Persian Medicine, Babol University of Medical Sciences, Babol, Iran; 11 Traditional Medicine and History of Medical Sciences Research Center, Health Research Institute, Babol University of Medical Sciences, Babol, Iran; 12 Community Medicine Department, Medical Faculty, Mazandaran University of Medical Sciences, Sari, Iran.; 13 Department of Persian Medicine, School of Medicine, Zanjan University of Medical Sciences, Zanjan, Iran; 14 Clinical Research Development Unit, Moradi Hospital in Rafsanjan University of Medical Sciences, Rafsanjan, Iran; 15 Department of Nursing, Zanjan Branch, Islamic Azad University, Zanjan, Iran; 16 Community Health Department, School of Nursing, Aja University of Medical Sciences, Tehran, Iran; 17 Institute for Health Sciences Research, Academic Center for Education, Culture and Research, ACECR, Tehran, Iran

**Keywords:** Iranian traditional medicine, Persian medicine, Temperament, Mizaj, Stomach, Su-e-Mizaj

## Abstract

**Background:**

Gastric disorders are one of the most common human ailments, which impose a huge economic burden on countries. In Persian Medicine (PM), it is possible to predict the susceptibility to gastric diseases with diagnosis of gastric Mizajes (temperaments) and dystemperaments. The semiology of gastric dystemperaments has been investigated in PM textbooks, although the value of each sign and symptom is not mentioned. Consequently, this research is designed to determine the major and minor criteria for classifying gastric dystemperaments on the basis of valid manuscripts and with the help of PM specialists in the present era.

**Methods:**

This was a consensus-based study consisting of four phases. In the first phase, reference PM textbooks were studied. Symptoms and signs of gastric dystemperaments were collected and listed in four groups. In the second phase, semi-structured interviews with a sample of PM experts were carried out. Phase three included a focused group discussion with experts. Eventually, findings were integrated from the three study phases in a two-day meeting in Sari City.

**Results:**

Selected criteria included eight major and eight minor criteria for hot-cold dystemperament, as well as six major and eight minor criteria for wet-dry gastric dystemperament.

**Conclusion:**

Modern lifestyles and the interfering factors are responsible for some changes in diagnostic signs and symptoms according to PM. This was the first step to coordinate PM diagnostic criteria for gastric dystemperaments. Further studies are recommended to reach a unique protocol in the field of PM diagnostics. The next step includes design and validation of national diagnostic tools.

Gastric disorders are one of the most common human sicknesses, with significant impact on health care services due to high prevalence. Not only do these diseases force a huge economic burden on countries, but they also rank second in drug expenditure (1–4). In the recent years, the World Health Organization (WHO) has decided to expand the use of complementary and traditional medicine according to the lack of public access to new health services and the trend to use complementary medical modalities in its international strategy (5). Persian medicine (PM) is one of the oldest medical schools in the world. PM scholars such as Abubakr Muhammad ibn Zakariyya al-Razi (Rhazes), Ali ibn Abbas Majusi Ahvazi (Haly Abbas), Ibn Sina (Avicenna), Jorjani and others have written many books on the diagnosis and classification of diseases.

Similar to most traditional approaches, including Traditional Chinese Medicine and Indian Ayurveda, they diagnosed and treated diseases based on individual differences. (6–13). According to PM principles, individual differences are presented in the context of Mizaj (temperament) (14–17). Mizaj is a qualitative concept which results from the ‘interaction of various elements existing in the human body, and influences the physical and mental state, as well as organ functions’. Based on the underlying principles, nine categories of Mizaj exist. While each person has a unique Mizaj based on the physical, functional and psychological characteristics, all Mizaj belong to one of these nine Mizaj categories (16–21). 

An inappropriate lifestyle may lead to deviation of any of these categories to an abnormal range to cause *Su-e-Mizaj* (dystemperament) (18, 22, 23). PM scholars believed that each body organ has a specific Mizaj to perform the special function effectively. Therefore, if the Mizaj of an organ becomes abnormal for any reason, it will not be able to perform its function properly and as a result, such organs will gradually have susceptibility to an ill condition called *‘Su-e-Mizaj’* or ‘dystemperament’ (22, 23).

As an important clinical principle in PM, the stomach is the main digestive organ with the health of other organs depending on its balance in four qualities (warm, cold, wet and dry). The symptoms of functional dyspepsia are consistent with some symptoms of gastric dystemperament. Functional dyspepsia can lead to other gastric diseases including gastric ulcer, irritable bowel syndrome and cancers. Therefore, the semiology of gastric dystemperaments can help us predict the susceptibility to some diseases, including the ones mentioned, before their onset (24, 25). As a complementary method, modifying gastric dystemperament will help us prevent specific ailments (26, 27). 

Unfortunately, previous studies on the topic had limited focus on a few PM textbooks in introducing signs of gastric dystemperaments (28). Furthermore, no systematic review using PM resources could be identified to specify the importance of each sign and symptom in diagnosing gastric dystemperaments distinctively (29, 30). For the first time, Alizadeh et al. (2017) presented a protocol for diagnosis of gastric diseases using PM books and expert opinions. However, without mentioning which particular sign or symptom is related to which gastric dystemperamt, they only listed a general presentation of signs and symptoms in their article. In addition, they did not mention how they used expert opinions and how they carried out the survey (30). Determining the value of each sign and symptom will play a major role in diagnosing a particular gastric disease and its treatment. Hoseinzadeh et al. (2018) presented a 59-item questionnaire including checklists and self-report questions for the diagnosis of gastrointestinal dystemperaments. In this study, gastric dystemperament indices and the weight of each in determining gastric dystemperaments are not specifically mentioned (31). 

To facilitate the use of PM viewpoints in gastric dystemperament diagnosis in practice and research, standard methods or tools are needed (29). Thus, the current study was conducted using PM textbooks, interviews with specialists, focused group discussion and finally, expert panels to define major and minor criteria for identifying gastric dystemperaments as a prerequisite access to the standard tools. 

## Methods

**Design and the study phases: **This was a consensus-based study consisting of four phases. In brief, phase I was a review of selected ancient literature to collect information on the symptoms and signs of gastric dystemperaments. To achieve this goal, one or two important textbooks from each century were selected and reviewed after consultation with the research team. Phase II included interviews with selected PM experts to extract signs and symptoms based on clinical experiences. Selection of experts was accomplished based on their interest, willingness to participate, gender and years of experience. 

With the coordination of each Persian medicine department, an expert with the entry criteria of at least 5 years of clinical experience and interested in collaborating in this research was invited. Interviews continued until data saturation was reached. Each interview lasted at least for one hour. All interviews were recorded and transcribed. Phase III was a focused group discussion with experts to provide a platform to ensure all symptoms of hot, cold, wet, and dry dystemperaments had been listed. Phase IV was a two-day meeting in Sari*, *North of Iran.

**The Sari meeting: **A group of experts were invited to participate in a two-day scientific meeting in Sari to integrate findings from the three study phases. Invitation of participants was based on their interests and experiences on Persian medicine. A member of the research team chaired the meetings and the main investigator was responsible to provide a report for each session. The final report was tabulated to summarize findings on the major and minor criteria for gastric dystemperaments.

**Ethical considerations: **Ethical issues have been completely observed by the research team, and the study has been conducted in accordance with COPE (Committee on Publication Ethics) guidelines. The Ethics Committee of Shahid Beheshti University of Medical Sciences approved this study with code IR.SBMU.RETECH.REC.1395-627.

## Results

The study period was from 2016 to 2018. Twelve experts took part in Sari meeting. The characteristics of experts are shown in table 1. 

According to the findings of phase I, II and III, there are twelve types of gastric dystemperaments. All dystemperaments were classified into three main groups: “simple dystemperaments” (*Su-e-Mizaj-e-Sada*) including hot, cold, dry and wet dystemperaments; “non-material compound dystemperaments” (*Su-e-Mizaj-e-Murakkab*), classified into four groups (hot-dry, hot-wet, cold-dry, cold-wet); and “compound-material dystemperaments” (*Su-e-Mizaj-e-Maddi*) comprising dominance of humors -phlegm, blood, yellow bile or black bile (20, 23, 32). 

The ancient PM scholars did not mention the major and minor criteria for diagnosing these diseases; however, they are mentioned in phase II and III, based on expert opinions in their clinical experiences. Signs and symptoms are scored differently in each group of dystemperament due to the importance of the four properties (hotness, coldness, wetness, and dryness). In the Sari meeting (expert panel) phase, the major and minor criteria for determining warm, cold, wet and dry dystemperaments were extracted according to results from previous phases and opinions of experts. The experts selected eight major and eight minor criteria for hot-cold gastric dystemperament, and six major and eight minor criteria for the wet-dry gastric dystemperament. Other signs and symptoms mentioned in textbooks did not get an acceptable score to be considered as criteria (tables 2-3).

According to the results, the most prominent indices for a hot gastric dystemperament include good gastric digestion, low appetite, increased thirst, red-colored tongue, feeling of hotness in the stomach (burning), dry stools, bitter taste in the mouth, fasting nausea, oral cavity rashes, dry mouth, disagreement with hot-natured foods and benefit from cold-natured foods. The stomach with a cold dystemperament shows some of these signs and symptoms: dyspepsia, increased appetite, bloating, white and milky color of the tongue, disagreement with cold-natured foods and benefit from hot-natured foods. 

The main signs and symptoms of wet gastric dystemperament include: lack of thirst, hypersalivation, nausea, round and flabby tongue, soft stool, after-meal sleepiness, eye puffiness, fast transition time, disagreement with wet-natured foods and benefit from dry-natured foods. Individuals with a dry gastric dystemperament, have to drink a little amount of water frequently when thirsty, because if they drink more water they feel full, and may feel a splash sound after drinking (*Takhazkhoz)*. Other symptoms include dry mouth, dry stools, a dark, sharp and thin tongue, disagreement with dry-natured foods and benefit from wet-natured foods.

**Table 1:** Demographic data of the PM experts who participated in Sari meeting

	**Gender**	**Age (year)**	**Affiliation**	**Work Experience (year)**
1	Male	49	Assistant Professor, School of Traditional Medicine, Shahid Beheshti University of Medical Sciences	20
2	Male	47	Associate Professor, School of Traditional Medicine, Shahid Beheshti University of Medical Sciences	15
3	Female	47	Assistant Professor, School of Traditional Medicine, Shahid Beheshti University of Medical Sciences	14
4	Female	42	Assistant Professor, School of Traditional Medicine, Shahid Beheshti University of Medical Sciences	13
5	Female	41	Assistant Professor, School of Traditional Medicine, Shahid Beheshti University of Medical Sciences	15
6	Male	40	Assistant Professor, School of Traditional Medicine, Tehran University of Medical Sciences	10
7	Female	40	Assistant Professor, School of Traditional Medicine, Tehran University of Medical Sciences	11
8	Male	44	Assistant Professor, Traditional Medicine and History of Medical Sciences Research Center, Babol University of Medical Sciences	17
9	Female	35	Assistant Professor, Traditional Medicine and History of Medical Sciences Research Center, Babol University of Medical Sciences	8
10	Female	35	Assistant Professor, Traditional Medicine and History of Medical Sciences Research Center, Babol University of Medical Sciences	7
11	Male	45	Assistant Professor, Department of Persian Medicine, Shahed University of Medical Sciences	18
12	Female	41	Assistant Professor, School of Traditional Medicine, Hamedan University of Medical Sciences	10

**Table 2:** Major and minor criteria for warm and cold dystemperaments of stomach

**Sign or symptom **	**Focus group of cold (n=8p)**	**Focus group of warm (n=13p)**	**Cold in Interviews (n=15p)**	**Warm in Interviews (n=15p)**	**Sari meeting**
*Major*					
Gastric digestion			11 (73%)	9 (60%)	97%
Impression from foods			4 (27%)	5 (33%)	94%
Thirstiness			4 (27%)	8 (53%)	85%
Bloating or distention			15 (100%)	3 (20%)	76%
Tongue color and coating			3 (20%)	2 (13%)	73%
Burning sensation (hotness) in the stomach	-		0	0	70%
Reflux) sour stomach)	-		4 (27%)	2 (13%)	61%
Transition time			4 (27%)	1 (7%)	55%
*Minor*					
Appetite			9 (60%)	9 (60%)	70%
Stool consistency			4 (27%)	6 (40%)	64%
Taste of the mouth	-		3 (20%)	4 (27%)	55%
Belching			3 (20%)	5 (33%)	52%
Saliva	-		5 (33%)	7 (47%)	42%
Fasting nausea	-		0	0	27%
Rashes in the mouth	-	-	0	0	24%
Impressionable from climate	-	-	0	0	18%

() means it was mentioned as a criterion. (-) means it wasn’t mentioned as a criterion. The percentage of experts who agreed were not registered in focus group sessions, and only their final decisions for accepting or declining were mentioned.

**Table 3:** Major and minor criteria for wet and dry dystemperaments of stomach

**Sign or symptom**	**The expert panel of Wet (n=13p)**	**The expert panel of Dry (n=7p)**	**Wet in Interviews** **(n=15p)**	**Dry in Interviews (n=15p)**	**Sari meeting**
*Major*					
Thirstiness	Lack of thirst		4 (27%)	9 (60%)	100%
Impression from foods			8 (53%)	4 (27%)	93%
Saliva			9 (60%)	9 (60%)	87%
Reflux			1 (6%)	1 (6%)	77%
Bloating			8 (53%)	2 (13%)	73%
Tongue color and coating		-	5 (33%)	5 (33%)	70%
*Minor*					
Eye puffiness		-	1 (6%)	0	57%
Stomach discomfort (due to incomplete digestion)			4 (27%)	1 (6%)	57%
Transition time		-	3 (20%)	0	53%
After meal sleepiness		-	1 (6%)	0	50%
Stool consistency	soft	dry	5 (33%)	5 (33%)	47%
Tongue form			1 (6%)	1 (6%)	47%
Takhazkhoz (splash sound after drinking)	-		0	2 (13%)	33%
nausea		-	1 (6)%	0	30%

() means it was mentioned as a criterion. (-) means it was not mentioned as a criterion. The percentage of experts who agreed were not registered in focus group sessions, and only their final decisions for accepting or declining were mentioned.

)) means it increases () it decreases

## Discussion

According to PM, most organ disorders lie in the category of Mizaj disorders, the diagnosis of which is helpful for identification of susceptibility to certain diseases. In the subject of gastric diseases, many signs and symptoms are mentioned for the diagnosis of gastric dystemperaments. It is, therefore, important to evaluate the value of each sign and symptom for better diagnosis. According to the exclusivity of wet-dry to hot-cold dystemperament, it is necessary to distinguish between their major signs and symptoms. The preference and priority of differentiating indices of gastric dystemperaments has not been indicated in written PM resources. 

There have been a few recently published articles that can be compared with the findings of the current study. Alizadeh et al. (2017) described digestion power as a major criterion for diagnosing all four dystemperaments, but according to the method of this study, stomach digestion is only a major criterion for diagnosing cold-hot, and a minor one for wet-dry gastric dystemperament. Based on written resources and the opinion of experts, good digestion is an important sign of a hot stomach and dyspepsia is a common symptom of cold gastric dystemperament. In contrast, salivation and tongue color were minor criteria for diagnosing all four dystemperaments of stomach in their study, while in this study, PM experts described them as major ones for wet-dry gastric dystemperament. Appetite and stool consistency were major criteria in their study, but are minor ones in the current study as they do not differentiate diagnosis between four major dystemperaments. Some symptoms like taste and smell in the mouth do not attract the attention of the patients and they do not express them to their physician. Therefore, experts omitted them from the diagnostic criteria. The results of the current study show that some of the gastric dystemperament diagnostic indices are not of great interest to PM specialists, due to changes in the general lifestyle and social habits. Other symptoms are affected by many interfering factors. For example, facial skin color, pulse and the temperature on the stomach area are affected by sunlight and environment temperature. That is why some of the diagnostic criteria mentioned by ancient scholars are not usable and helpful today. Moreover, there was no strong evidence as of how much they had used these kinds of indices in practice. There is a great need for standard national diagnostic questionnaires and instruments to standardize diagnosis for Persian medicine or Unani medicine practitioners. 

**Limitations and advantages: **The results of the current study are the first step to coordinate PM practice nationwide regarding gastric dystemperament diagnosis. Despite the desire to finalize the exact major and minor criteria for gastric dystemperaments diagnosis, the Sari expert group postponed this crucial issue until more studies are conducted based on patient visits. This study, as the first to identify the major and minor indices of wet-dry and hot-cold stomach dystemperaments separately, more PM references were studied compared to previous studies. In addition, a larger number of the researchers from different universities throughout Iran participated in the study (15 PM physicians in the interview phase and 12 PM specialists in the Sari Meeting phase and 4 methodologists). However, limited access to typical dystemperamental diseases and the unwillingness of some experts to participate in the study led to a lack of information about different opinions of experts in this field. Therefore, it is suggested to perform more researches in the future by motivating and involving more patients and specialists.

In conclusion as the next step, it is recommended to design and validate standard national diagnostic tool for gastric dystemperament using our findings. Some people with mild gastric dystemperament symptoms, are asymptomatic, while prone to gastrointestinal diseases, such as dyspepsia, peptic ulcer, and stomach cancer. Thus, standard gastric dystemperament diagnostic tools will enable timely diagnosis and prevention. These diagnostic tools will enable us to provide an appropriate lifestyle plan for people according to their gastric temperament and take a step forward in the prevention and treatment of gastric diseases.
